# Ovarian stimulation in IVF couples with severe male factor infertility: GnRH antagonist versus long GnRH agonist

**DOI:** 10.3389/fendo.2022.1037220

**Published:** 2022-10-07

**Authors:** Mu Lv, Juanjuan Yu, Peiqin Chen, Qimeng Xiao, Liqun Lou, Yifan Luo, Mu Yuan, Yuan Xu, Youji Feng, Mingzhu Bai, Zhenbo Zhang, Linxia Li

**Affiliations:** ^1^ Department of Obstetrics and Gynecology, Reproductive Medicine Center, Shanghai General Hospital, Shanghai Jiao Tong University School of Medicine, Shanghai, China; ^2^ Department of Obstetrics and Gynecology, The International Peace Maternity & Child Health Hospital of China Welfare Institute, Shanghai Jiao Tong University School of Medicine, Shanghai, China; ^3^ Department of Obstetrics and Gynecology, Shanghai First Maternity and Infant Hospital, Tongji University School of Medicine, Shanghai, China; ^4^ Center for Reproductive Medicine, Maternal and Child Health Hospital in Xuzhou, Xuzhou, China; ^5^ Shanghai Key Laboratory for Assisted Reproduction and Reproductive Genetics, Renji Hospital, Shanghai Jiao Tong University School of Medicine, Shanghai, China; ^6^ Department of Obstetrics and Gynecology, Reproductive Medicine Center, Tongji Hospital, School of Medicine, Tongji University, Shanghai, China; ^7^ Department of Obstetrics and Gynecology, Seventh People’s Hospital of Shanghai University of Traditional Chinese Medicine, Shanghai, China

**Keywords:** *in vitro* fertilization, severe male factor infertility, ovarian stimulation, GnRH antagonist, long GnRH agonist, live birth rate

## Abstract

**Objective:**

To examine the efficacy of gonadotropin releasing hormone (GnRH) antagonist (GnRH-ant) protocol and the long GnRH agonist (GnRH-a) protocol during *in vitro* fertilization (IVF) therapy in patients with severe male infertile factors.

**Methods:**

A total of 983 women with severe male factor infertility undergoing IVF therapy from 2017 to 2020 at one center were retrospectively analyzed. Patients were divided into the GnRH-ant group (n=527) and the GnRH-a group (n=456) according to their ovarian stimulation protocols. Patient baseline characteristics, ovarian stimulation characteristics, and clinical pregnancy outcomes were compared between the groups. The live birth rate was considered the main pregnancy outcome.

**Results:**

GnRH-a group had a higher live birth rate compared with the GnRH-ant group (41.0% versus 31.3%, p=0.002). Moreover, the implantation (32.8% vs. 28.1%, p=0.033), biochemical pregnancy (52.4% versus 44.8%, p=0.017), clinical pregnancy (49.3% versus 39.7%, p=0.002) and ongoing pregnancy rates (43.2% vs. 34.9%, p=0.008) were higher in GnRH-a group. For patients with one embryo transferred, the GnRH-a group demonstrated higher live birth (37.0% vs. 19.4%, p=0.010) and ongoing pregnancy rate (38.9% vs. 24.5%, p=0.046) than the GnRH-ant group. Among patients with two embryos transferred, the live birth rate was also higher in the GnRH-a group than in the GnRH-ant group, with no statistical difference. No significant differences were observed in the biochemical abortion rate, clinical miscarriage rate, early miscarriage rate, late miscarriage rate, heterotopic pregnancy rate, twin pregnancy rate, and birth sex ratio between the two groups.

**Conclusion:**

For individuals with severe male infertility undergoing IVF, the GnRH-a protocol is considered a more efficient and feasible strategy with a higher live birth rate compared to the GnRH-ant protocol, especially in single embryo transfer.

## Introduction

Infertility is presently defined as one year of unplanned non-conception with unprotected intercourse throughout the fertile part of the menstrual cycle (1). Infertility affects 9% of couples globally (2), with a male factor accounting for 50% of the reasons for infertile couples (3). Male infertility is frequently caused by sperm deficits, which are manifested as reduced spermatogenesis, sperm DNA damage, loss of sperm motility, and aberrant sperm morphology (4, 5). Different factors affect sperm production, ultimately leading to oligozoospermia, asthenozoospermia, teratozoospermia, or a combination of these symptoms (oligoasthenoteratozoospermia [OAT]) (6). Severe male factor infertility includes severe oligozoospermia, cryptozoospermia, azoospermia, severe asthenospermia and severe teratospermia (7, 8).

Patients with male factor infertility were once thought to be sterile. Male factor infertility research shows that it may be successfully handled using *in vitro* fertilization, resulting in high implantation and pregnancy rates (9). The advancements in assisted reproductive technology (ART), particularly the invention of intracytoplasmic sperm injection (ICSI), testicular sperm aspiration (TESA), and microdissection testicular sperm extraction (micro-TESE), have radically altered this scenario (10–12). Epidemiological studies have shown that spouses of men with azoospermia are likely to be younger and have a better ovarian reserve (13). Because these couples receive ART treatment early in life, their response to ovarian stimulation is usually positive, resulting in an adequate fertility rate after IVF procedures. Therefore, it is critical to evaluate which ovarian stimulation approach is optimal for these women.

Ovarian stimulation is an important element of the IVF technique because it enables the selection of high-quality embryos for transfer (14). Long GnRH agonist protocol and GnRH antagonist protocol are two commonly used ovarian stimulation regimens. Other ovulation stimulation protocols include mild stimulation protocol, short agonist protocol and progestin primed ovarian stimulation (PPOS) protocol (14). GnRH agonists have been applied since the early 1980s, and their role in controlled ovarian stimulation is significant (15). The standard long GnRH agonist is widely used because of its stable and higher clinical pregnancy rate in the fresh embryo transfer of IVF patients (16). Recently, GnRH antagonist regimen has gained popularity and has received attention due to its short treatment time, low injection volume, and low incidence of ovarian hyperstimulation syndrome (OHSS) (17). Several retrospective studies have compared the effectiveness of these two regimens, but have shown inconsistent results (18–20). This study is the first to investigate the effectiveness and feasibility of GnRH antagonist protocol and long GnRH agonist protocol in patients with severe male factor infertility who underwent IVF. The findings of this study might help the doctors to choose an appropriate protocol for patients with severe male factor infertility.

## Materials and methods

### Participants

**Figure 1:**
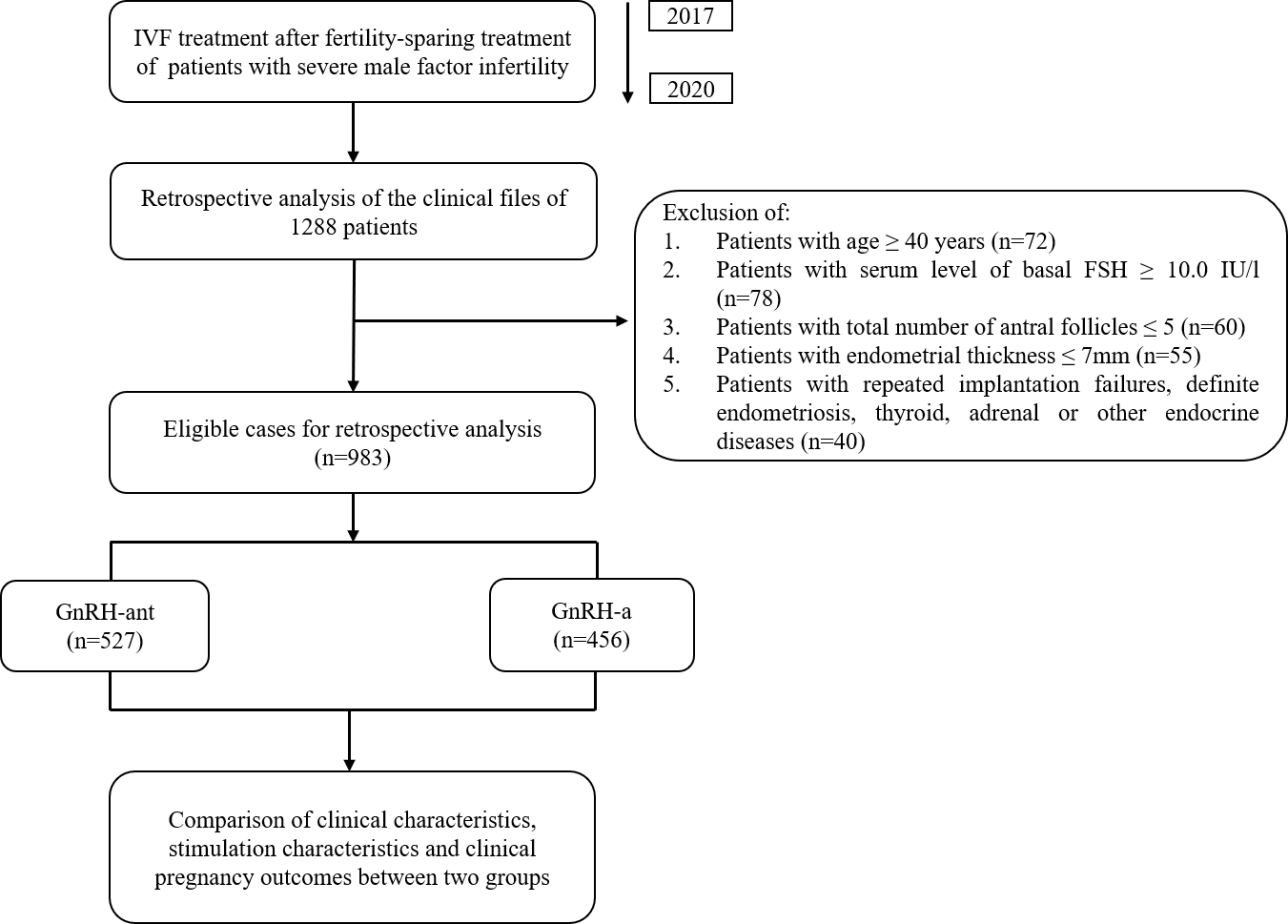
Flowchart summarizing the design of the present retrospective study.

This retrospective study was performed at the Reproductive Medicine Center of Shanghai General Hospital between 1 January 2017 and 31 December 2020. Inclusion criteria: (1) Patients undergoing their first IVF cycles; (2) Infertility caused by severe male factor; (3) Received GnRH-ant protocol or GnRH-a protocol for their treatment. Exclusive criteria: (1) age ≥ 40 years (n=72); (2) serum level of basal follicle stimulating hormone (FSH) ≥10.0 IU/L (n=78); (3) total number of antral follicles ≤ 5 (n=60); (4) endometrial thickness before embryo transfer ≤ 7mm (n=55); (5) patients with repeated implantation failures, definite endometriosis, thyroid, adrenal or other endocrine diseases (n=40). The overall design of the current study was shown in **Figure 1**. In this study, we selected patients with severe male infertility for study by assessing semen quality according to the fifth edition of the WHO guideline (6). All patients were properly advised of the associated risks of IVF therapy and completed an informed permission form to allow researchers to utilize their clinical data. The principles of the Helsinki Declaration are followed in this investigation.

### Ovulation stimulation protocols

In the GnRH-ant group, 150-225IU recombinant FSH (Merck, US) or HMG (Lizhu, China) was administered subcutaneously or intramuscularly daily on the second or third day of the menstrual cycle, depending on the BMI, age, and ovarian reserve of patients (21, 22). Follicular development was observed as detailed below, and the gonadotrophin dosage was adjusted according to the monitored follicular growth rate. Cetrorelix (0.25 mg, Merck, Swiss) was introduced when the maximal follicular diameter reached 14 mm or luteinizing hormone (LH) levels exceeded 10 IU/L.

In the GnRH-a group, vaginal B-ultrasonography was conducted on the second day of menstruation for individuals who had a normal menstrual cycle. When there was no ovarian cystic structure ≥ 2 cm, Diphereline (3.75mg, Ipsen Pharma Biotech) was injected. On the 28th day following a Diphereline injection, blood FSH, LH, estradiol (E2), progesterone (P), and vaginal B-ultrasound were measured. The stimulation of gonadotrophins was started with either recombinant FSH (Gonal-F, Merck, Geneva, Swiss) or HMG (Lizhu, Zhuhai, China). Based on age, antral follicle count, and baseline FSH levels, the initial dosage varied from 150IU to 300IU (21, 22).

When the B-ultrasound examination reveals that three dominant follicles are ≥ 17 mm in diameter and the blood E2 level of each dominant follicle reaches 800 pmol/L, discontinue the injection of recombinant FSH/HMG for injection, human chorionic gonadotropin (HCG) 6000 IU was injected at 8:30 p.m. and eggs were harvested after 34 to 36 hours.

### Oocyte retrieval, IVF, and fresh embryo transplantation

Each follicle was aspirated individually, and the follicular fluid containing oocytes was collected. The cumulus oocyte complex was evaluated using the known oocyte maturation score criteria. ICSI was performed on MII oocytes 4h after oocyte retrieval. Embryos were cultured until day 3, at which point all available embryos were evaluated. A high-quality embryo was chosen for transfer. Grade I-II embryos with 7-12 cells at the cleavage stage were selected (23–25). Under ultrasound guidance, one or two fresh embryos were transferred. Supernumerary embryos were cryopreserved using vitrification in accordance with the criteria established by Cummins et al. (26).

### Pregnancy test

A Serum HCG test was performed 14 days after the embryo transfer, and the vaginal ultrasound was done 28 days following the embryo transfer. HCG positivity was defined as HCG levels of more than 5 IU/L. Clinical pregnancy refers to the presence of a gestational sac in the uterus (27). All pregnant patients were followed up until they gave birth or miscarried.

### Outcome measures

The primary outcome was the live birth rate, which was defined as the ratio of live birth cycles to all cycles. The secondary outcome measures were implantation rate, biochemical pregnancy rate, biochemical abortion rate, clinical pregnancy rate, clinical miscarriage rate, early miscarriage rate, late miscarriage rate, ongoing pregnancy rate and heterotopic pregnancy rate, twin pregnancy rate, and birth sex ratio. Implantation rate is the ratio of the total number of gestational sacs to the number of embryos transferred. Biochemical pregnancy is defined as 14 days after embryo transfer, the HCG level in pregnant women was > 5 IU/L. Biochemical abortion is defined as HCG positive in blood 14 days after embryo transfer but no gestational sac detected 28 days after transfer. Clinical pregnancy refers to the discovery of a gestational sac in the uterus 28 days after embryo transfer. Miscarriages that occur before 14 weeks of pregnancy are called early miscarriages, and those after 14 weeks are called late miscarriages. Ongoing pregnancy is defined as the pregnancy lasting until 20 weeks or later.

### Statistical analysis

SPSS 25.0 software was used for statistical analysis (IBM Corp., Armonk, NY, USA). The Kolmogorov-Smirnov test was used for the normality test. The Student’s t-test was used to compare the data that were normally distributed, and the results were expressed by mean ± standard deviation (SD). Man-Whitney U test was used when data were not normally distributed and the results were expressed by median (first quartile, third quartile). Categorical variables were analyzed using the Chi-squared test and the results were expressed by percentage (%). Comparisons between GnRH-ant group and GnRH-a group were performed using the Man-Whitney U test and chi-square tests. p<0.05 was considered statistically significant.

### Ethics statement

This study was reviewed and approved by the Institutional review board and ethics committee of Shanghai General Hospital (NO.2022SQ414). The participants provided their written informed consent to participate in this study.

## Results

This research comprised 527 women who underwent GnRH-ant ovarian stimulation and 456 women who underwent GnRH-a ovarian stimulation. **Table 1** shows the basic information about patient characteristics. The results of the normality test showed that all the measurement data in the present study were non-normally distributed. Baseline clinical characteristics between the GnRH-ant and GnRH-a groups were similar and no significant differences were observed in maternal age, body mass index, duration of infertility, basal LH, basal E2, and type of infertility (p>0.05).

**Table 1:** Clinical features of patients in different ovarian stimulation groups.

Group	GnRH-ant (n = 527)	GnRH-a (n = 456)	*P*-value
Characteristic			
Maternal Age (years)	29 (27, 31)	30 (27, 33)	0.609
Body mass index (kg/m^2^)	20.76 (19.53, 23.03)	22.31 (20.5, 24.61)	0.466
Duration of infertility (years)	3 (2, 4)	3 (2, 5)	0.621
Basal LH (IU/L)	6.60 (5.45, 7.76)	6.36 (5.43, 7.50)	0.466
Basal E2 (pmol/L)	103 (43.13, 175.25)	102 (61, 173)	0.304
Type of infertility, n (%)			0.825
Primary infertility	392/527 (74.4%)	342/456 (75.0%)	
Secondary infertility	135/527 (25.6%)	114/456 (25.0%)	

Data are presented as the M(P25~P75) for continuous variables and n (%) for categorical variables.

As shown in **Table 2**, the duration of gonadotropin (Gn) treatment, total Gn dosage, endometrial thickness, number of oocytes obtained, the proportion of MII oocytes, fertilization rate, and number of embryos transferred were substantially higher in the GnRH-a group than in the GnRH-ant group (p<0.05). Furthermore, there was no significant difference in average Gn dosage, peak E2 level at trigger day, and P level at trigger day between the two ovulation stimulation regimens (p>0.05).

**Table 2:** Stimulation characteristics for each ovarian stimulation group.

Characteristic	GnRH-ant (n = 527)	GnRH-a (n = 456)	*P*-value
Duration of Gn treatment (days)	8 (7, 9)	10 (9, 12)	<0.001
Total Gn dosage (IU)	2025 (1575, 2400)	2400 (1500, 3300)	0.003
Average Gn dosage (IU)	235.36 (200, 300)	238.75 (220, 300)	0.226
Peak E2 level at trigger day (pmol/L)	9730.50 (6699.50, 13386.00)	9701.00 (6693.50, 15058.79)	0.273
P level at trigger day (nmol/L)	2.87 (1.86, 3.84)	2.11 (1.52, 3.46)	0.202
LH level at trigger day (IU/L)	2.98 (1.83, 4.80)	1.67 (0.80, 3.39)	<0.001
Endometrial thickness (mm)	9.8 (9, 11.8)	10 (9, 12)	0.032
Number of oocytes obtained	13 (9, 17)	15 (13, 17)	<0.001
proportion of MII oocytes	4640/7070 (65.6%)	4169/4829 (86.3%)	<0.001
fertilization rate	4275/7070 (60.5%)	3471/4829 (71.9%)	<0.001
number of embryos transferred	2 (1, 2, 1.74 ± 0.439)	2 (2, 2, 1.89 ± 0.319)	<0.001

Data are presented as the M(P25~P75, mean ± SD) for the average number of embryos transferred, and n (%) for categorical variables.

MII, metaphase II.

As for pregnancy outcomes (**Table 3** **)**, the GnRH-a group demonstrated higher live birth than the GnRH-ant group (41.0% vs. 31.3%, p=0.002, OR 1.525, 95%CI 1.173~1.982). In addition, implantation rate (32.8% vs. 28.1%, p=0.033, OR 1.247, 95%CI 1.018~1.527), biochemical pregnancy rate (52.4% vs. 44.8%, p=0.017, OR 1.358, 95%CI 1.056~1.746), clinical pregnancy rate (49.3% vs. 39.7%, p=0.002, OR 1.482, 95%CI 1.150~1.909), ongoing pregnancy rate (43.2% vs. 34.9%, p=0.008, OR 1.418, 95%CI 1.096~1.835) were significantly higher in the GnRH-a group than that in the GnRH-ant group. For patients with one embryo transferred, the GnRH-a group demonstrated higher ongoing pregnancy rate (38.9% vs. 24.5%, p=0.046, OR 1.965, 95%CI 1.006~3.841) and live birth (37.0% vs. 19.4%, p=0.010, OR 2.440, 95%CI 1.219~4.884) than the GnRH-ant group (**Supplemental Table 1**). Although not statistically different, among patients who transferred two embryos, the GnRH-a group demonstrated higher live birth than the GnRH-ant group (**Supplemental Table 2**). No differences were observed in the biochemical abortion rate, clinical miscarriage rate, early miscarriage rate, late miscarriage rate, heterotopic pregnancy rate, twin pregnancy rate, and birth sex ratio between the two groups (p>0.05).

**Table 3:** Comparison of the treatment outcomes for patients with severe male factor infertility.

Outcome	GnRH-ant (n = 527)	GnRH-a (n = 456)	P-value	OR (95%CI)
Implantation rate	257/915 (28.1%)	281/858 (32.8%)	0.033	1.247 (1.018~1.527)
Biochemical pregnancy rate	236/527 (44.8%)	239/456 (52.4%)	0.017	1.358 (1.056~1.746)
Biochemical abortion rate	27/527 (5.1%)	14/456 (3.1%)	0.108	0.587 (0.304~1.133)
Clinical pregnancy rate	209/527 (39.7%)	225/456 (49.3%)	0.002	1.482 (1.150~1.909)
Clinical miscarriage rate	25/209 (12.0%)	28/225 (12.4%)	0.878	1.046 (0.588~1.860)
Early miscarriage rate	21/209 (10.0%)	27/225 (12.0%)	0.517	1.221 (0.667~2.234)
Late miscarriage rate	4/209 (1.9%)	1/225 (0.4%)	0.152	0.229 (0.025~2.064)
Ongoing pregnancy rate	184/527 (34.9%)	197/456 (43.2%)	0.008	1.418 (1.096, 1.835)
Heterotopic pregnancy rate	8/527 (1.5%)	5/456 (1.1%)	0.564	0.719 (0.234~2.214)
Twin pregnancy rate	48/209 (23.0%)	55/225 (24.4%)	0.718	1.085 (0.697~1.690)
Live birth rate	165/527 (31.3%)	187/456 (41.0%)	0.002	1.525 (1.173~1.982)
Birth sex ratio	95: 104	114: 110	0.517	1.135 (0.774~1.662)

Data are presented as n (%) for categorical variables.

Birth sex ratio is expressed as male to female ratio.

95%CI, 95% Confidence Interval; OR, Odds Ratio.

## Discussion

The present study demonstrated that the GnRH-a protocol was more effective than the GnRH-ant protocol for patients with severe male factor infertility in terms of higher live birth rates. The implantation rates, biochemical pregnancy, clinical pregnancy, and ongoing pregnancy rates were also higher in the GnRH-a group. Moreover, in patients with one embryo transferred, the GnRH-a group demonstrated a higher live birth rate and ongoing pregnancy rate than the GnRH-ant group. Therefore, the GnRH-a protocol is a better choice for patients with severe male factor infertility undergoing IVF, especially in single embryo transfer.

A high live birth rate is a common goal for both patients and physicians. In order to avoid some confounding factors of frozen cycles, we only selected fresh cycles to analyze the pregnancy outcomes of the two regimens. We found that the live birth rate, implantation rate, biochemical pregnancy rate, clinical pregnancy rate, and ongoing pregnancy rate were higher in the GnRH-a group than in the GnRH-ant group. These results suggest that the GnRH-a regimen has better endometrial receptivity in patients with severe male factor infertility. However, when the population of single embryo transfer and double embryo transfer is counted separately, there was no difference in the implantation rate, biochemical pregnancy rate and clinical pregnancy rate, which may be due to the small sample size of each group, resulting in low statistical efficiency. Although the advantage of the GnRH-a protocol reduced when 2 embryos transferred, the GnRH-a protocol is still a better option for patients with severe male infertility because the GnRH-a protocol had significantly better pregnancy outcomes than the antagonist protocol. In China, in order to improve the success rate of each fresh embryo transfer, the GnRH-a protocol is still regarded as the main ovulation induction protocol (28, 29). A meta-analysis showed that the live birth rate was on average 1.5% higher with the GnRH-a regimen than with the GnRH-ant regimen (30). Ruan et al. found that the physiological secretory function of the endometrium was restored after the application of the GnRH-a regimen, and the uterine receptivity was improved (31). A comparative proteomic analysis showed that the GnRH-ant protocol impaired endometrial receptivity more severely than the GnRH-a regimen (32). The above findings suggest that the GnRH-a regimen has better endometrial receptivity compared with the GnRH-ant regimen. Besides, we found the number of oocytes, the MII rate, and the fertilization rate in the GnRH-a regimen were greater than in the GnRH-ant regimen, suggesting that the GnRH-a regimen facilitated the maturation of the ovum, follicular maturity, and fertilization ability.

However, because the long GnRH agonist regimen over-inhibited the pituitary and lowered pituitary responsiveness in the early stages of ovulation promotion, the long GnRH agonist regimen increased the usage of Gn to different degrees and prolonged the use time of Gn (33). GnRH antagonist protocol, discovered in the 1990s, can competitively inhibit GnRH receptors, resulting in a fast reduction of Gn release, and greatly shortening the treatment time (34, 35). GnRH antagonists have some advantages for individuals with decreased ovarian reserve (DOR) (36). Studies have demonstrated that for DOR patients, a GnRH antagonist regimen can achieve similar clinical results as a GnRH agonist regimen, with shorter medication duration, more patient acceptability, and a decreased risk of OHSS (17). In our study, the GnRH-ant regimen shortened the stimulation period and reduced the Gn dosage compared with the long protocol. Besides, the GnRH-ant protocol had a lower clinical miscarriage rate and twin pregnancy rate compared to the GnRH-a protocol but no statistical difference, which was consistent with previous report (37). In terms of the birth sex ratio, the long regimen had a higher proportion of male births, while the antagonist regimen had a higher proportion of female births, but there was no statistical difference. This may be due to the small number of patients and the unavoidable heterogeneity of individuals in various regimens. However, what’s important is that our results suggested that the GnRH-ant regimen had lower implantation rates and lower live birth rates in patients with severe male factor infertility, possibly because of lower endometrial receptivity, which was consistent with previous studies (38, 39).

This study is limited by retrospective design. Patients were assigned to two ovulation stimulation regimens based on physician judgment and patient selection; therefore, selection bias is possible and potential confounding factors could not be explained. This retrospective study was conducted in only one reproductive center. Additional large-scale randomized controlled trials are needed to confirm the conclusion of this study. The molecular mechanism of GnRH-a and GnRH-ant regimens leading to different pregnancy outcomes in patients with severe male factor infertility needs to be further studied.

In conclusion, the findings of the study demonstrated that the GnRH-a protocol is a more efficient ovulation stimulation method for patients with severe male infertility. Therefore, improvement of the live birth rate with the aid of GnRH-a protocol may improve the long-term prognosis of patients with severe male infertility undergoing IVF, especially in single embryo transfer.

## Data availability statement

The raw data supporting the conclusions of this article will be made available by the authors, without undue reservation.

## Ethics statement

The studies involving human participants were reviewed and approved by the Ethics Committee of Shanghai General Hospital (NO.2022SQ414). The participants provided their written informed consent to participate in this study.

## Author contributions

LLi and ZZ contributed to the conception or design of the work. MB contributed to the revision of the manuscript for important intellectual content. ML, JY and PC contributed to the acquisition, analysis of the work. QX, LLo, YL, MY, YX and YF contributed to the interpretation of data for the work. All authors contributed to the article and approved the submitted version.

## Funding

This work was supported by grants from the National Natural Science Foundation of China (grants 881902630, 8187211, 81672562 and 81902630), grants from the National Key Technology R&D Program of China (2019YFC1005200 and 2019YFC1005201), Shanghai Municipal Science and Technology Committee of Shanghai outstanding academic leaders plan (19XD1423100), the project of Outstanding Medical Doctor for ZZ, Shanghai Municipal Education Commission—Gaofeng Clinical Medicine Grant Support (20181714).

## Conflict of interest

The authors declare that the research was conducted in the absence of any commercial or financial relationships that could be construed as a potential conflict of interest.

## Publisher’s note

All claims expressed in this article are solely those of the authors and do not necessarily represent those of their affiliated organizations, or those of the publisher, the editors and the reviewers. Any product that may be evaluated in this article, or claim that may be made by its manufacturer, is not guaranteed or endorsed by the publisher.
