# Supplementary material for: Ovarian stimulation in IVF couples with severe male factor infertility: GnRH antagonist versus long GnRH agonist
Source: Front Endocrinol (Lausanne). 2022 Oct 7;13:1037220. doi: 10.3389/fendo.2022.1037220 (PMC9585245; doi:10.3389/fendo.2022.1037220)
Supplement: Supplementary file 1 [file DataSheet_1.docx]

**Supplemental Table 1** Comparison of the treatment outcomes for patients with one embryo transferred.

| Outcome | GnRH-ant (n=139) | GnRH-a (n=54) | P-value | OR (95%CI) |
| --- | --- | --- | --- | --- |
| Implantation rate | 37/139 (26.6%) | 22/54 (40.7%) | 0.056 | 1.895 (0.979~3.668) |
| Biochemical pregnancy rate | 44/139 (31.7%) | 23/54 (42.6%) | 0.152 | 1.602 (0.839~3.059) |
| Biochemical abortion rate | 7/139 (5.0%) | 1/54 (1.9%) | 0.319 | 0.356 (0.043~2.962) |
| Clinical pregnancy rate | 37/139 (26.6%) | 22/54 (40.7%) | 0.056 | 1.895 (0.979~3.668) |
| Clinical miscarriage rate | 3/37 (8.1%) | 1/22 (4.5%) | 0.599 | 0.540(0.053~5.534) |
| Early miscarriage rate | 3/37 (8.1%) | 1/22 (4.5%) | 0.599 | 0.540 (0.053~5.534) |
| Late miscarriage rate | 0/37 (0.0%) | 0/22 (0.0%) |  |  |
| Ongoing pregnancy rate | 34/139 (24.5%) | 21/54 (38.9%) | 0.046 | 1.965 (1.006~3.841) |
| Heterotopic pregnancy rate | 1/139 (0.7%) | 0/54 (0.0%) | 0.532 | 0.993 (0.979~1.007) |
| Twin pregnancy rate | 0/37 (0.0%) | 0/22 (0.0%) |  |  |
| Live birth rate | 27/139 (19.4%) | 20/54 (37.0%) | 0.010 | 2.440 (1.219~4.884) |

*Data are presented as n (%) for categorical variables.*

*95%CI, 95% Confidence Interval; OR, Odds Ratio.*

**Supplemental Table 2** Comparison of the treatment outcomes for patients with two embryos transferred.

| Outcome | GnRH-ant (n=388) | GnRH-a (n=402) | P-value | OR (95%CI) |
| --- | --- | --- | --- | --- |
| Implantation rate | 220/776 (28.4%) | 259/804 (32.2%) | 0.095 | 1.201 (0.969~1.489) |
| Biochemical pregnancy rate | 192/388 (49.5%) | 216/402 (53.7%) | 0.232 | 1.185 (0.897~1.568) |
| Biochemical abortion rate | 20/388 (5.2%) | 13/402 (3.2%) | 0.177 | 0.615 (0.302~1.254) |
| Clinical pregnancy rate | 172/388 (44.3%) | 203/402 (50.5%) | 0.083 | 1.281 (0.968~1.695) |
| Clinical miscarriage rate | 22/172 (12.8%) | 27/203 (13.3%) | 0.884 | 1.046 (0.572~1.913) |
| Early miscarriage rate | 18/172 (10.5%) | 26/203 (12.8%) | 0.482 | 1.257 (0.664~2.380) |
| Late miscarriage rate | 4/172 (2.3%) | 1/203 (0.5%) | 0.123 | 0.208 (0.023~1.878) |
| Ongoing pregnancy rate | 150/388 (38.7%) | 176/402 (43.8%) | 0.144 | 1.236 (0.930, 1.641) |
| Heterotopic pregnancy rate | 7/388 (1.8%) | 5/402 (1.2%) | 0.520 | 0.685 (0.216~2.178) |
| Twin pregnancy rate | 48/172 (27.9%) | 55/203 (27.1%) | 0.860 | 0.960 (0.609~1.513) |
| Live birth rate | 138/388 (35.6%) | 167/402 (41.5%) | 0.085 | 1.287 (0.966~1.716) |
| Birth sex ratio | 80:92 | 100:104 | 0.628 | 1.106 (0.737~1.660) |

*Data are presented as n (%) for categorical variables.*

*Birth sex ratio is expressed as male to female ratio.*

*95%CI, 95% Confidence Interval; OR, Odds Ratio.*
